# Peroxisome proliferator-activated receptor α (PPARα) mRNA expression in human hepatocellular carcinoma tissue and non-cancerous liver tissue

**DOI:** 10.1186/1477-7819-9-167

**Published:** 2011-12-15

**Authors:** Tsuyoshi Kurokawa, Yoshiharu Shimomura, Gustavo Bajotto, Katsuhiro Kotake, Takashi Arikawa, Nobuhiro Ito, Akira Yasuda, Hiroshi Nagata, Toshiaki Nonami, Kazuo Masuko

**Affiliations:** 1 Masuko Memorial Hospital and Institute for Medical Research, Takehashi-cho, 35-26, Nakamura-ku, Nagoya, Japan; 2 Department of Materials Science and Engineering, Shikumi College, Nagoya Institute of Technology, Furo-cho, Chikusa-ku, Nagoya, Japan; 3 Department of Surgery, Aichi Medical University, Yazako-Karimata 21, Nagakute-cho, Aichi, Japan

**Keywords:** hepatocellular carcinoma, PPARα, cyclin D1, CPT1A, energy metabolism; carcinogenesis

## Abstract

**Background:**

Peroxisome proliferator-activated receptor α (PPARα) regulates lipid metabolism in the liver. It is unclear, however, how this receptor changes in liver cancer tissue. On the other hand, mouse carcinogenicity studies showed that PPARα is necessary for the development of liver cancer induced by peroxisome proliferators, and the relationship between PPARα and the development of liver cancer have been the focus of considerable attention. There have been no reports, however, demonstrating that PPARα is involved in the development of human liver cancer.

**Methods:**

The subjects were 10 patients who underwent hepatectomy for hepatocellular carcinoma. We assessed the expression of PPARα mRNA in human hepatocellular carcinoma tissue and non-cancerous tissue, as well as the expression of target genes of PPARα, carnitine palmitoyltransferase 1A and cyclin D1 mRNAs. We also evaluated glyceraldehyde 3-phosphate dehydrogenase, a key enzyme in the glycolytic system.

**Results:**

The amounts of PPARα, carnitine palmitoyltransferase 1A and glyceraldehyde 3-phosphate dehydrogenase mRNA in cancerous sections were significantly increased compared to those in non-cancerous sections. The level of cyclin D1 mRNA tends to be higher in cancerous than non-cancerous sections. Although there was a significant correlation between the levels of PPARα mRNA and cyclin D1 mRNA in both sections, however the correlation was higher in cancerous sections.

**Conclusion:**

The present investigation indicated increased expression of PPARα mRNA and mRNAs for PPARα target genes in human hepatocellular carcinoma. These results might be associated with its carcinogenesis and characteristic features of energy production.

## Background

Peroxisome proliferator-activated receptors (PPARs) are extremely diverse metabolic regulators of mainly glycerolipids [1,2]. They are nowadays thought to play a central role in energy homeostasis in response to changes in the environment [3]. In recent years there have also been reports that PPARs have antiinflammatory properties [4,5] and contribute to the control of cell proliferation signals and apoptosis [6,7]), so that they have become a focus of considerable attention with regard to metabolic diseases such as diabetes and hyperlipidemia, as well as severe diseases including inflammatory disorders and cancer.

The energy metabolism of cancer cells is known to have peculiar characteristics that differ from those of normal cells, although the details remain sketchy. Among PPARs, peroxisome proliferator-activated receptor α (PPARα) is found mainly in the liver, where it is known to regulate lipid metabolism [8]. It is unclear, however, how PPARα and other factors are associated with metabolic changes in liver cancer tissues, and the question of the kinds of changes that occur in energy metabolism as liver cells become cancerous is one of great interest.

From carcinogenesis experiments using mice it is well known that PPARα is necessary for development of liver cancer induced by peroxisome proliferators [9], and the relationship between PPARα and the development of liver cancer has been the focus of considerable attention. There have been no reports, however, on findings in human liver cancer.

In the present study we assessed the expression of PPARα mRNA in human hepatocellular carcinoma (HCC) tissues and non-cancerous tissues, as well as the expression of target genes of PPARα, carnitine palmitoyltransferase 1A (CPT1A) and cyclin D1 mRNAs, to clarify one part of the relationship between carcinogenesis and the energy metabolism characteristics of liver cancer cells. We also evaluated glyceraldehyde 3-phosphate dehydrogenase (G3PDH), a key enzyme in the glycolytic system in both tissues.

## Materials and methods

The subjects were 10 patients at our institution who underwent hepatectomy for HCC. Samples from cancerous and non-cancerous sections of the resected specimens were quickly frozen and stored at -80°C until they were used for measurement. Only samples for which the diagnosis of HCC had been confirmed pathologically were used. Background liver had chronic hepatitis or cirrhosis, and the level of the cirrhosis was A or B in Child's classification.

The current study was reviewed and approved by the ethical review board at Aichi Medical University. Informed consent was obtained from each patient.

### Reverse transcription and PCR amplification

Total RNA from a sample of about 90 mg was isolated by a modified guanidinum isothiocyanate method [10] with the ISOGEN reagent (Nippon Gene Co. Ltd., Tokyo, Japan). RNA concentrations were spectrophotometrically determined. First-strand cDNA synthesis of 5 μg total RNA was performed using oligo(dT) primers following instructions of the manufacturer; SuperScript First-Strand Synthesis System for RT-PCR, (Invitrogen Life Technologies, Carlsbad, California, USA). Reverse transcription for 18S rRNA analysis was carried out using random hexamers. Amplifications of the target cDNAs were performed using the following synthetic oligonucleotides obtained from Rikaken Co., Ltd. (Nagoya, Japan).

PPARα: forward 5'-CCAGTATTTAGGAAGCTGTCC-3' and reverse 5'-AAGTTCTTCAAGTAGGCCTCG-3';

CPT1A: forward 5'-AGACGGTGGAACAGAGGCTGAAG-3' and reverse 5'-TGAGACCAAACAAAGTGATGATGTCAG-3';

G3PDH: forward 5'-AACAGCGACACCCACTCCTC-3' and reverse 5'-CCAGGAAATGAGCTTGACAA;

Cyclin D1: forward 5'-CCGTCCATGCGGAAGATC-3' and reverse 5'-ATGGCCAGCGGGAAGAC-3';

18S rRNA: forward 5'-CGACGACCCATTCAAAAATC-3' and reverse 5'-AACCCTGATTCCCCATCAC-3'.

The primer sets yield products of 432, 81, 492, 86, and 90 base pairs for CPT1A, G3PDH, PPARα, cyclin D1, and 18S rRNA, respectively. PCR reactions (25 μl final volume) were carried out in a TaKaRa PCR Thermal Cycler model TP3000 (TaKaRa Biomedicals, Ohtsu, Japan) using the following cycle conditions: denaturation at 94°C for 30 sec, annealing for 50 sec, and extension at 72°C for 50 sec. Annealing temperatures were 58, 54, 54, 60, and 56°C for CPT1A, G3PDH, PPARα, cyclin D1, and 18S rRNA, respectively. The numbers of cycles for amplification of CPT1A, G3PDH, PPARα, cyclin D1, and 18S rRNA templates were 28, 23, 29, 35, and 35, respectively, as confirmed to be within the linear range of amplification in preliminary experiments. PCR products (10 μl reaction mixture) were run on 1.5% agarose gels and visualized by ethidium bromide staining. Signals on the gels were visualized by UV transillumination, the gels were photographed, and the images were analyzed by scanning densitometry using the Scion Image Beta 4.02 software (Scion Corporation, Frederick, Maryland, USA).

mRNA levels for CPT1A, G3PDH, PPARα, and cyclin D1 in each sample were normalized by the amount of 18S rRNA.

### Statistical analysis

Results were expressed as means ± SEM. Comparison of the difference between the mean values of two groups was made by the Mann-Whitney's U test. Correlation between expression levels of PPARα and cyclin D1 mRNAs was obtained with the Spearman's rank correlation test. A difference with a *P *value of < 0.05 was considered statistically significant.

## Results

### Gene expression analysis

**Figure 1:**
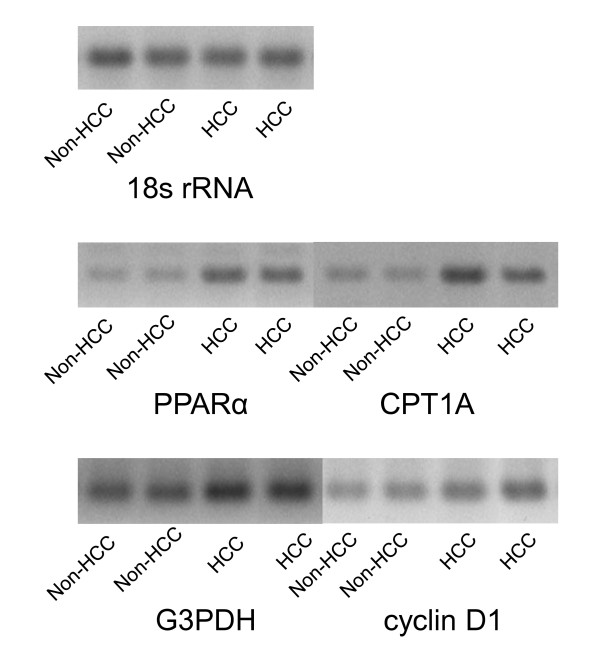
**Representative photographs of each PCR product in cancerous and non-cancerous sections**. Representative photographs of PCR products of 18s rRNA, PPARα, CPT1A, G3PDH and Cyclin D1 mRNAs in cancerous and non-cancerous sections were shown.

Representative photographs of PCR products of 18s rRNA, PPARα, CPT1A, G3PDH and Cyclin D1 mRNAs in cancerous and non-cancerous sections were shown in Figure 1.

Comparison of expression levels of PPARα, CPT1A, G3PDH and cyclin D1 mRNAs in cancerous and non-cancerous sections

**Figure 2:**
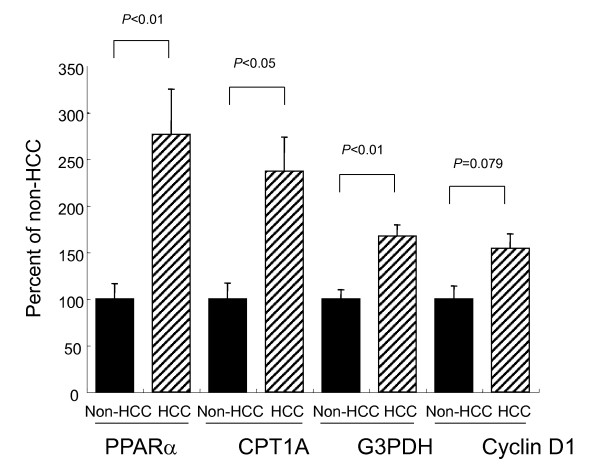
**Comparison of expression levels of PPARα, CPT1A, G3PDH and cyclin D1 mRNAs in cancerous and non-cancerous sections**. Values are expressed as the ratio of the mRNA amount in cancerous sections to that in non-cancerous sections. Amounts of PPARα, CPT1A and G3PDH mRNAs in cancerous sections were significantly higher, compared to those in non-cancerous sections. The level of cyclin D1 mRNA tended to be higher in cancerous sections than in non-cancerous sections, although the difference did not reach a statistically significant level.

Figure 2 shows the levels of PPARα, CPT1A, G3PDH and cyclin D1 mRNA expression in cancerous and non-cancerous sections. The values are expressed as a ratio of the mRNA amount in cancerous sections to that in non-cancerous sections. The amounts of PPARα and CPT1A mRNAs in cancerous sections were 277 ± 49% and 237 ± 36%, respectively, compared to those in non-cancerous sections. These were statistically significant differences (*P *< 0.01 and *P *< 0.05, respectively). The amount of G3PDH mRNA in cancerous sections was 167 ± 12%, compared to that in non-cancerous sections. This was also statistically significantly different (*P *< 0.01). The level of cyclin D1 mRNA tended to be higher in cancerous sections (154 ± 16%) than in non-cancerous sections, although the difference did not reach a statistically significant level (*P *= 0.079). The levels of 18S rRNA in non-cancerous and cancerous sections were nearly the same, at 100.0 ± 5.0% and 97.3 ± 5.3%, respectively.

**Figure 3:**
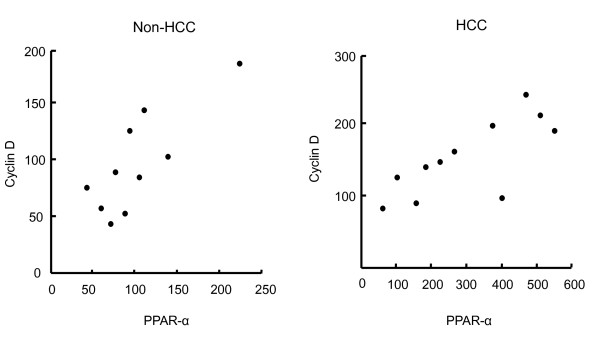
**Correlation between expression levels of PPARα and cyclin D1 mRNAs in non-cancerous sections and cancerous sections**. A significant correlation was seen in non-cancerous sections and cancerous sections. The regression coefficient and *P *value of non-cancerous sections were 0.80 and 0.004 and those of cancerous sections were 0.74 and 0.006, respectively.

Figure 3 shows the correlation between expression levels of PPARα and cyclin D1 mRNAs in non-cancerous sections and cancerous sections. A significant correlation was seen in non-cancerous sections and cancerous sections; the regression coefficient and *P *value of non-cancerous sections were 0.80 and 0.004 and those of cancerous sections were 0.74 and 0.006, respectively.

Level of 18S rRNA, the internal standard, in non-cancerous sections and cancerous sections

The levels of 18S rRNA in non-cancerous and cancerous sections were nearly the same, at 100.0 ± 5.0% and 97.3 ± 5.3%, respectively.

## Discussion

Cancer cells have a characteristic energy production system which differs from that in normal cells. The 18-Fluoro-2-deoxyglucose (FDG) positron emission tomography (FDG-PET), which is recently employed in diagnosing the presence of cancer and treatment monitoring, also takes advantage of these characteristics in cancer cells [11]. In general, the glucose metabolism is increased in malignant tumors, owing to increased levels of glucose transporter proteins and increased levels of intracellular enzymes that promote glycolysis, such as hexokinase and phosphofructokinase. In most malignant cells, the relatively low levels of glucose-6-phosphate lead to accumulation and trapping of FDG intracellularly, allowing the visualization of increased FDG uptake compared with normal cells. However, there are several types of cancer that have low visualization rates of the focus with FDG-PET [12,13]. Compared with other cancers, these cancers are presumed to have different characteristics in their energy metabolism. HCC is included in this low visualization rate group [14].

The liver is the main target organ of PPARα metabolic action. Activation of PPARα in liver cells induces expression of acyl-CoA synthase, CPT1A 8 (the rate-limiting enzyme of fatty acid β-oxidation system) and other proteins necessary for the transport of fatty acids into the mitochondria, the site of the ß-oxidation system [15,16]. The results of this study suggest that the expression of PPARα mRNA is upregulated in HCC tissues compared with non-cancerous tissues. Results similar to those for PPARα were also obtained for CPT-1A mRNA expression.

It is generally said that sugar metabolism is highly enhanced in cancer tissues. With the aim of assessing the state of sugar metabolism in normal and cancerous liver cells, we evaluated the abundance of mRNA of G3PDH, a key enzyme in the glycolytic system. The level of G3PDH mRNA was certainly increased in cancerous sections. However, we previously reported a comparison of the expression of pyruvate dehydrogenase kinase (PDK) mRNA in HCC tissue and non-cancerous tissue [17]. In this report, there tended to be less PDK in HCC tissues than in non-cancerous tissues. PDK inactivates the pyruvate dehydrogenase complex (PDC), which is a key enzyme in systems that produce energy by glucose oxidation [18]. Therefore, PDK plays an important role in regulation of glucose oxidation. These results suggest that glucose oxidation might play a less important role in energy production in HCC tissues compared with other cancer tissues.

There is a possibility that lipids rather than carbohydrates might be mainly used in the energy production in HCC tissue. Thus, one reason there is not a high visualization rate of HCC on FDG-PET may be that, in comparison with other types of cancer, there is less use of carbohydrates in HCC cells.

PPARα has been a focus of recent attention in terms of its relationship with carcinogenesis. Long-term administration of peroxisome proliferators in mice leads to the development of HCC [9], but in PPARα knock-out mice this phenomenon is not seen [19]. PPARα may be understood to function as an oncogene in this model. In addition, continuous activation of PPARα increases the expression of cell cycle regulatory factors such as cyclin and cyclin-dependent kinase, and inhibits apoptosis [7]. Tatsumi et al. [20] demonstrated activation of RXRα, a heterodimer partner of PPARα, in HCV core protein transgenic mice, and speculated that PPARα contributes to the development of human HCC. The cyclin D1 encodes the regulatory subunit of a holoenzyme that phosphorylates and inactivates the Rb protein and promotes progression through G1 to S phase of the cell cycle. Over expression of cyclin D1 plays important roles in the development of human cancers, including breast, melanoma and prostate [21-23]. In 1993, Zhang et al. [24] has indicated the amplification and over expression of cyclin D1 in HCC. Reducing the expression of cyclin D1 reportedly promotes the apoptosis of HCC in vitro [25,26]. We also indicated that the level of cyclin D1 mRNA was increased in HCC tissues. Moreover, a very strong correlation between PPARα and cyclin D1 expression levels was seen in HCC tissues. This indicates that in tissue with enhanced expression of PPARα, the expression of cyclin D1 will also be enhanced. Activation of PPARα reportedly contributes to the carcinogenesis process for liver cancer, and the results of this study indicate the possibility that this may also hold true for humans. However, as is often pointed out, increases in peroxisome proliferation and incidence of HCC have not been demonstrated in patients who take peroxisome proliferators long term [27], so we cannot say with certainty that PPARα contributes to the development of liver cancer in humans.

## Conclusions

The present investigation indicated increased expression of PPARα mRNA and mRNAs for PPARα target genes in human hepatocellular carcinoma. These results might be associated with its carcinogenesis and characteristic features of energy production, but further study is warranted.

## Abbreviations list

PPARα: peroxisome proliferator-activated receptor α; CPT1A: carnitine palmitoyltransferase 1A; G3PDH: glyceraldehyde 3-phosphate dehydrogenase; HCC: hepatocellular carcinoma.

## Competing interests

The authors declare that they have no competing interests.

## Authors' contributions

TK carried out design of the study and drafted manuscript. YS and GB carried out experimental procedures, data analysis, and reviewed the paper. KK, TA, NI, AY and HN were involved in the collection of surgical specimens. TN and KM guaranteed the whole study. All authors read and approved the final manuscript.
